# Physical Isolation and Mental Health Among Older U.S. Adults in the COVID-19 Coping Study

**DOI:** 10.1093/geroni/igab046.145

**Published:** 2021-12-17

**Authors:** Carly Joseph, Brendan O'Shea, Jessica Finlay, Lindsay Kobayashi

**Affiliations:** 1 Central Michigan University, Ann Arbor, Michigan, United States; 2 University of Michigan, Ann Arbor, Michigan, United States

## Abstract

The ongoing COVID-19 pandemic has set an urgent need to understand the impact of physical isolation on mental health. We aimed to investigate the relationships between physical isolation during the period when many US states had shelter-in-place orders (April-May 2020) and subsequent longitudinal trajectories of mental health in middle-aged and older adults (aged 55+, N=3,978) over a six-month follow-up (April to October 2020). We used population and attrition-weighted multivariable linear mixed-effects models. At baseline, 7 days/week of physical isolation (vs. 0 days/week) was associated with elevated depressive symptoms (β=0.82; 95% CI: 0.04-1.60), and all of 1-3, 4-6, and 7 days/week of physical isolation (vs. 0 days/week) were associated with elevated anxiety symptoms and loneliness. Physical isolation was not associated with changes in mental health symptoms over time. These findings highlight the need to prioritize opportunities for in-person connection for middle-aged and older adults when safe to do so.

